# Impact of the COVID-19 outbreak on out-of-hospital cardiac arrest management and outcomes in a low-resource emergency medical service system: a perspective from Thailand

**DOI:** 10.1186/s12245-022-00429-1

**Published:** 2022-06-09

**Authors:** Sattha Riyapan, Jirayu Chantanakomes, Pakorn Roongsaenthong, Parinya Tianwibool, Borwon Wittayachamnankul, Jirapong Supasaovapak, Wasin Pansiritanachot

**Affiliations:** 1 Department of Emergency Medicine, Faculty of Medicine Siriraj Hospital, Mahidol University, 2 Wanglang Road, Bangkok Noi, Bangkok, Thailand 10700; 2 Siriraj Emergency Medical Services Center, Siriraj Hospital, Bangkok, Thailand; 3 Department of Emergency Medicine, Faculty of Medicine, Chiang Mai University, Chiang Mai, Thailand; 4 Department of Emergency Medicine, Rajavithi Hospital, Department of Medical Services, Ministry of Public Health, Bangkok, Thailand

**Keywords:** COVID-19, Emergency medical services, Out-of-hospital cardiac arrest

## Abstract

**Background:**

The impact of the coronavirus disease 2019 (COVID-19) outbreak on out-of-hospital cardiac arrest (OHCA) has been of interest worldwide. However, evidence from low-resource emergency medical service systems is limited. This study investigated the effects of the COVID-19 outbreak on the prehospital management and outcomes of OHCA in Thailand.

**Methods:**

This multicentered, retrospective, observational study compared the management and outcomes of OHCA for 2 periods: pre-COVID-19 (January–September 2019) and during the outbreak (January–September 2020). Study data were obtained from the Thai OHCA Network Registry. The primary outcome was survival rate to hospital discharge. Data of other OHCA outcomes and prehospital care during the two periods were also compared.

**Results:**

The study enrolled 691 patients: 341 (49.3%) in the pre-COVID-19 period and 350 (50.7%) in the COVID-19 period. There was a significant decrease in the survival rate to discharge during the COVID-19 outbreak (7.7% vs 2.2%; adjusted odds ratio [aOR], 0.34; 95% confidence interval [CI], 0.15–0.95). However, there were no significant differences between the 2 groups in terms of their rates of sustained return of spontaneous circulation (33.0% vs 31.3%; aOR, 1.01; 95% CI, 0.68–1.49) or their survival to intensive care unit/ward admission (27.8% vs 19.8%; aOR, 0.78; 95% CI, 0.49–1.15). The first-responder response interval was significantly longer during the COVID-19 outbreak (median [interquartile range] 5.3 [3.2–9.3] min vs 10 [6–14] min; *P* < 0.001). There were also significant decreases in prehospital intubation (66.7% vs 48.2%; *P* < 0.001) and prehospital drug administration (79.5% vs 70.6%; *P* = 0.024) during the COVID-19 outbreak.

**Conclusion:**

There was a significant decrease in the rate of survival to hospital discharge of patients with OHCA during the COVID-19 outbreak in Thailand. Maintaining the first responder response quality and encouraging prehospital advanced airway insertion might improve the survival rate during the COVID-19 outbreak.

## Introduction

Since 2020, severe acute respiratory syndrome coronavirus 2 (SARS-CoV-2) has spread worldwide and resulted in the coronavirus disease 2019 (COVID-19) outbreak [1, 2]. The COVID-19 outbreak directly causes fatalities and impacts other health outcomes, especially in emergency conditions [3–5]. The health system worldwide has faced a shortage of resources and personnel and consequent delays in providing emergency care [3, 5]. During lockdown periods, outpatient schedules were postponed, resulting in the progression of many diseases [6, 7]. Out-of-hospital cardiac arrest (OHCA) is one of the conditions that has been affected by overburdened health care systems [8]. For instance, a layperson may feel frightened to provide bystander cardiopulmonary resuscitation (CPR). In addition, OHCA management protocols have been modified to protect health care providers from COVID-19 infection. For example, emergency medical service (EMS) providers must now wear appropriate personal protective equipment (PPE). If they are not properly attired, the EMS and emergency department (ED) personnel have been instructed to avoid aerosol-generating procedures, such as intubation [9]. However, such new practices may increase EMS response intervals.

Research revealed the impact of the COVID-19 outbreak during 2020 on the chain of survival of OHCA relative to the normal situation [8]. Studies found that while some countries experienced decreased bystander CPR rates [10, 11], others did not [12, 13]. The research also identified a decrease in resuscitation attempts by EMS personnel [11], delayed EMS response intervals [14, 15], and a decrease in intubation rates during the outbreak period [12, 16, 17]. Furthermore, return of spontaneous circulation (ROSC), survival rate to hospital discharge, and survival with favorable neurological outcomes decreased during the outbreak in several countries [12, 15, 18–20]. However, most recent data were from high-income countries with well-developed EMS systems. To our knowledge, no data have been presented for low-resource EMS systems. Although there has been no consensus on categorizing EMS resource settings, with different environment of resuscitation practice and limited financial resources unlike those in high-income countries, Thai EMS systems have been categorized in the low-resource setting [21]. Therefore, this study investigated the effects of the COVID-19 outbreak on OHCA management and outcomes in a low-resource EMS system using data drawn from Thailand’s OHCA Network Registry.

## Methods

### Study design, setting, and population

This retrospective observational study drew upon data for the years 2019 and 2020 from the Thai OHCA Network Registry. The Registry was established under the auspices of the Thai Resuscitation Council in 2018. The Registry aims to promote the use of cardiac arrest data to improve the outcomes of OHCA in the community. During the study period, 3 hospitals participated in the network. Two of the hospitals are in Bangkok, the capital city of Thailand. One is located in a predominantly suburban area, whereas the other is in a commercial urban area of Bangkok. The third hospital is in the city of Chiang Mai in northern region of Thailand. Before the current study started, its protocol was approved by the respective institutional review board of each of the 3 participating hospitals.

The EMS system at each participating site, as detailed in Table 1, was a two-tier response system. It consisted of volunteer-based ambulances for basic life support (BLS) teams and hospital-based ambulances funded by the government for advanced life support (ALS) teams. All providers are trained and certified by the National Institute of Emergency Medicine of Thailand. The ALS team leaders including physicians, paramedics, or emergency nurse practitioners are qualified for conducting advanced cardiac life support and essential life-saving procedures such as advanced airway insertion, and CPR drug administration. All ALS teams followed 2015 American Heart Association Guidelines for Cardiopulmonary Resuscitation.

**Table 1:** Characteristics of each EMS participating site

Participating site	Urban capital	Suburban capital	Regional
City	Bangkok	Bangkok	Chiang Mai
Service area population	1,200,000	103,800	50,000
Population density (per km^2^)	15,000	8650	8300
Ambulance:population ratio	1:68,100	1:50,000	1:25,000
Annual number of EMS call	1200	900	750
Type of providers			
ALS	Physician, ENP, EMT-B	ENP, EMT-B	Physician, paramedic, nurse, EMT
BLS	EMT-B or EMR	EMT-B or EMR	EMT or EMR

*Abbreviations*: *ALS* advanced life support, *BLS* basic life support, *EMS* emergency medical services, *EMT* emergency medical technician, *EMT-B* emergency medical technician-basic, *EMR* emergency medical responder, *ENP* emergency nurse practitioner

In the event of an OHCA response, the dispatch center would simultaneously deploy a BLS team as the first responder and an ALS team to the scene. In addition, the dispatcher would initially provide dispatcher-assisted CPR to bystanders. The BLS team provided chest compression and bag-valve-mask ventilation using a reservoir system connected to an oxygen tank and applied an automatic external defibrillator. When the ALS team arrived, it provided high-quality CPR, manual defibrillation for shockable rhythms, vascular access, advanced airway insertion, and CPR drug administration. All participating sites applied the same universal termination of resuscitation criteria to withhold resuscitation. The characteristics of the EMS systems of the participating sites were described in a previous study [22].

During the COVID-19 outbreak in 2020, the participating sites changed their prehospital OHCA management protocols, as did other countries. Specifically, the EMS and ED providers were required to don appropriate PPE. Two participating sites allowed their providers to perform endotracheal intubations at the scene as the ALS team included a physician. One site recommended that EMS providers avoid advanced airway insertion during the COVID outbreak. In the ED, providers performed early endotracheal intubation using video laryngoscopes. They were also encouraged to use mechanical CPR devices to reduce the number of resuscitation providers.

### Data sources, data collection, and selection of study participants

The participating hospitals developed the OHCA Network Registry using variables and definitions from the Pan-Asian Resuscitation Outcome Study [23]. The Registry was based on the Utstein template guidelines for reporting OHCA [24]. Each site was responsible for collecting data and controlling their quality. The data were on patient characteristics; cardiac arrest location; prehospital details (provision of bystander CPR, public automatic external defibrillator use, initial rhythm, prehospital advanced airway insertion, and drug administration); initial rhythm in the ED; and patient outcomes. The details were initially recorded on paper by EMS providers or the emergency physicians, and the data were later entered into the Web registry (https://webapps2.duke-nus.edu.sg/eparos/index.jsp). Study investigators reviewed the quality of the data before exporting them for analysis.

The study included adults (age ≥ 18 years) with non-traumatic OHCA. EMS utilized patients, defined as OHCA patients who received prehospital resuscitation by EMS providers, and OHCA patients who were privately transported to the hospital were both eligible in this study. Patients who had Do Not Resuscitate orders were excluded. Eligible participants were assigned to a pre-COVID-19 group (patients experiencing OHCA January–September 2019) and a COVID-19 group (patients experiencing OHCA January–September 2020). A “public location” was defined as health care facilities, public and commercial buildings, nursing homes, streets and highways, industrial places, transportation centers, and recreation places. “First responder” was the BLS team dispatched by an emergency call center but not transporting the patient. “Prehospital advanced airway” included endotracheal intubation and supraglottic airway devices (SGA), that is, laryngeal mask airways) and laryngeal tubes. Lastly, “sustained ROSC” was defined as the return of pulse for more than 20 consecutive minutes.

### Outcome measurements

The primary objective of this study was to compare the survival-to-discharge rates of the pre-COVID-19 and COVID-19 groups. The secondary outcomes were all other OHCA outcomes, such as sustained ROSC at ED and survival to intensive care units (ICU) or wards admission. The study also compared the prehospital management performance of the 2 groups: bystander CPR, EMS response interval, first-responder response interval, prehospital intubation, and prehospital drug administration.

### Statistical methods

Based on previous data of the registry, approximately 7% of the patients with OHCA would survive to hospital discharge in the pre-COVID-19 period, while 2.2% survived to hospital discharge during the COVID-19 outbreak. Using a type I error of 5% and a power of 80%, a minimum sample size of 339 participants per group was required. Descriptive analyses of demographic data, clinical characteristics, prehospital management, post-resuscitation care, and patient outcomes were performed in all eligible OHCA patients and also in the EMS utilized subgroup. The categorical variables of the 2 groups were compared using a chi-square test or Fisher’s exact test. The prehospital interval was presented as the median and interquartile range and compared using the Mann–Whitney *U* test. We illustrated the prehospital intubation and drug administration rates of both study groups and the incidence of COVID-19 by month in a run chart. The OHCA outcomes were analyzed using multiple logistic regression adjusted for age, initial cardiac arrest rhythm, bystander CPR, and witnessed status. The study also analyzed the OHCA outcomes of the shockable-first-rhythm subgroup. The results of the analyses are presented as odds ratios (ORs) and 95% confidence intervals (CIs). Probability (*P)* values of < 0.05 were considered statistically significant. PASW Statistics for Windows, version 18.0 (SPSS Inc., Chicago, IL, USA) was used for all data analyses.

## Results

**Fig. 1:**
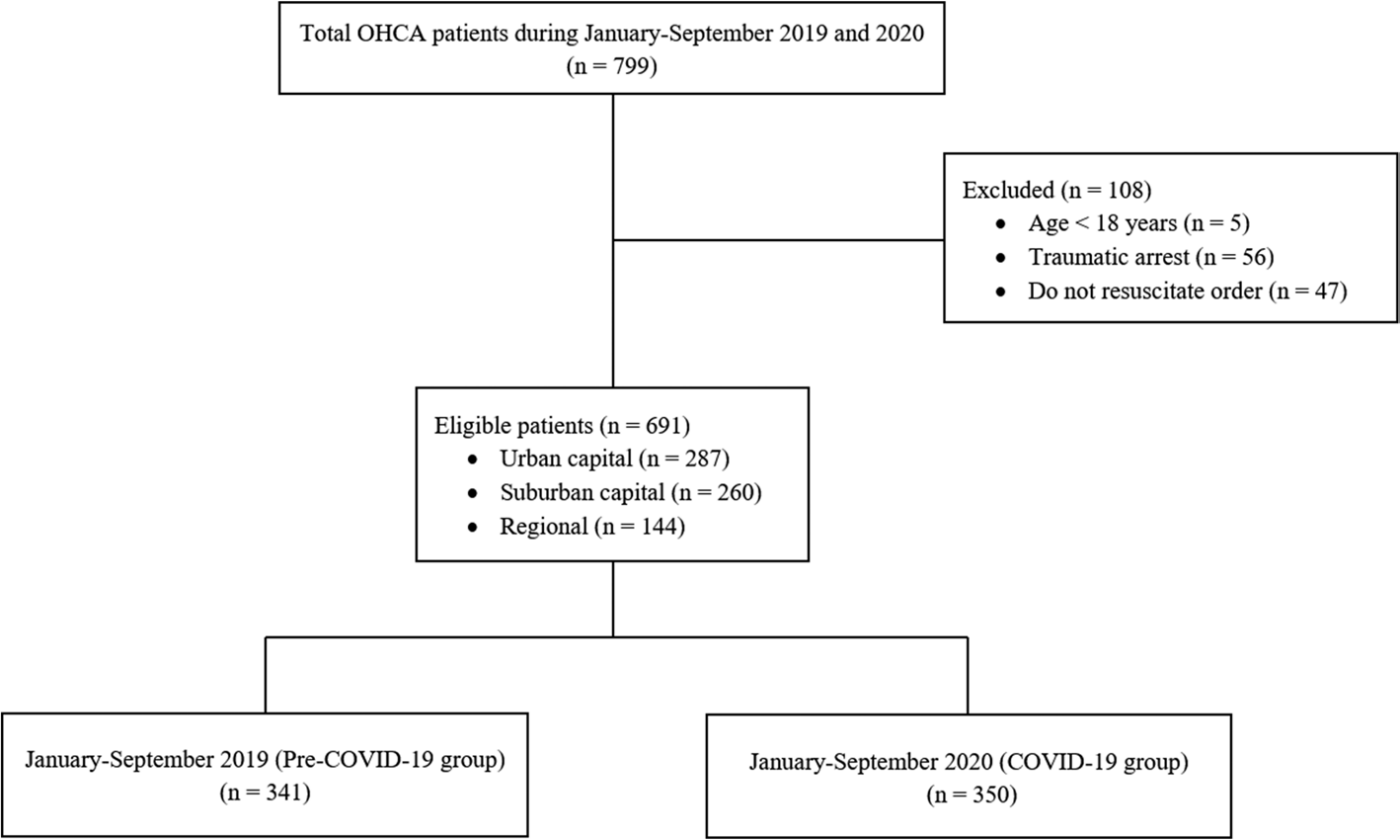
Data flow of patients in the study

The data flow of the research is illustrated in Fig. 1. From 799 patients in the OHCA Network Registry during January–September 2019 and January–September 2020, there were 108 patients excluded from the study: 5 for age under 18 years, 56 for traumatic arrest, and 47 for having a do-not-resuscitate order. Ultimately, the study enrolled 691 patients. Of these, 287 (41.5%) were from a predominantly suburban area of Bangkok, and another 260 (37.6%) were from a primarily commercial area. In addition, 144 (20.8%) patients were from the city of Chiang Mai in northern Thailand. We assigned 341 (49.3%) patients to the pre-COVID-19 group and 350 (50.7%) to the COVID-19 group.

Table 2 details the characteristics of the patients with OHCA in this study. Missing data on each variable ranged from 3.8% for the first arrest rhythm to 21.3% for bystander CPR. The differences between the pre-COVID-19 and COVID-19 groups were mainly nonsignificant. There was a significant higher proportion of hyperlipidemia in the COVID-19 outbreak period (7.9% vs 13.1%; *P* = 0.025).

**Table 2:** Characteristics of out-of-hospital cardiac arrest of pre-COVID-19 and COVID-19 outbreak groups

Characteristics	Total (***N*** = 691) ***n*** (%)	Pre-COVID-19 outbreak (2019) (***n*** = 341) ***n*** (%)	COVID-19 outbreak (2020) (***n*** = 350) ***n*** (%)	***P*** value
**Age, mean (SD), years**	63.07 (18.9)	62.7 (18.5)	63.4 (19.4)	0.387
**Age > 65 years**	352 (50.9)	164 (48.1)	188 (53.7)	0.140
**Male**	418 (60.5)	210 (61.6)	208 (59.4)	0.562
**Past medicine history**				
Heart disease	122 (17.7)	52 (15.2)	70 (20)	0.102
Diabetes	161 (23.3)	79 (23.2)	82 (23.4)	0.935
Hypertension	232 (33.6)	113 (33.1)	119 (34)	0.810
Respiratory	51 (7.4)	28 (8.2)	23 (6.6)	0.410
Hyperlipidemia	73 (10.6)	27 (7.9)	46 (13.1)	0.025
Stroke	49 (7.1)	20 (5.9)	29 (8.3)	0.215
Others	266 (38.5)	132 (38.7)	134 (38.3)	0.909
Unknown	142 (20.5)	78 (22.9)	64 (18.3)	0.136
**EMS utilized**	515 (74.5)	254 (74.5)	261 (74.6)	0.980
**Location**				0.155
Home residence	498 (72.1)	239 (70.1)	259 (74)	
Public location	127 (18.4)	62 (18.2)	65 (18.6)	
Others	66 (9.6)	40 (11.7)	26 (7.4)	
**Arrest witnessed (missing = 63)**	403 (64.2)	199 (65.7)	204 (62.8)	0.448
**Bystander CPR (missing = 63)**	252 (40.1)	133 (43.9)	119 (36.6)	0.063
**Bystander using public AED (missing = 147)**	29 (5.3)	14 (5.2)	15 (5.5)	0.865
**First arrest rhythm (missing = 26)**				0.083
Asystole	381 (57.3)	188 (57.5)	193 (57.1)	
Ventricular fibrillation	65 (9.8)	26 (8)	39 (11.5)	
Ventricular tachycardia	5 (0.8)	3 (0.9)	2 (0.6)	
Pulseless electrical activity	133 (20.0)	71 (21.7)	62 (18.3)	
Unknown shockable rhythm	15 (2.3)	12 (3.7)	3 (0.9)	
Unknown unshockable rhythm	17 (2.6)	6 (1.8)	11 (3.3)	
Unknown	49 (7.4)	21 (6.4)	28 (8.3)	
**First arrest rhythm groups,** ***n*** **(%) (missing = 26)**				0.629
Shockable rhythm group	85 (12.8)	41 (12.5)	44 (13)	
Unshockable rhythm group	531 (79.8)	265 (81)	266 (78.7)	
Unknown	49 (7.4)	21 (6.4)	28 (8.3)	
**Cause of arrest (missing = 127)**				0.208
Presumed cardiac etiology	320 (56.7)	165 (61.1)	155 (52.7)	
Respiratory	140 (24.8)	62 (23)	78 (26.5)	
Electrocution	10 (1.8)	6 (2.2)	4 (1.4)	
Drowning	2 (0.4)	1 (0.4)	1 (0.3)	
Others	92 (16.3)	36 (13.3)	56 (19)	
**Post arrest care (missing = 39)**				
Emergency PCI performed	15 (2.3)	9 (2.7)	6 (1.9)	0.462
Hypothermia therapy initiated	5 (0.8)	4 (1.2)	1 (0.3)	0.187
ECMO therapy initiated	6 (0.9)	2 (0.6)	4 (1.2)	0.395

Abbreviations: *AED* automatic external defibrillator, *COVID-19* coronavirus disease 2019, *CPR* cardiopulmonary resuscitation, *ECMO* extracorporeal membrane oxygenation, *EMS* emergency medical service, *PCI* percutaneous coronary intervention, *SD* standard deviation

**Fig. 2:**
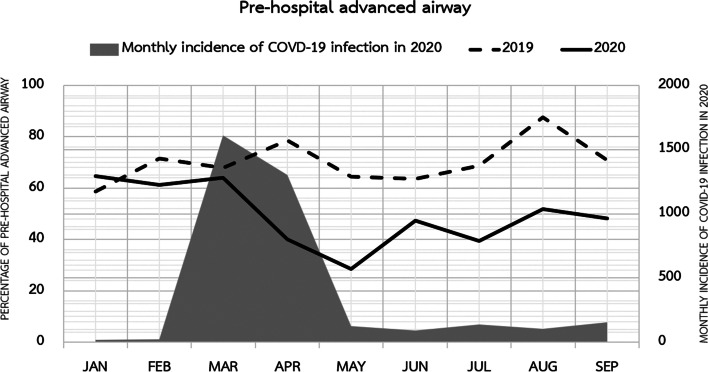
Incidence of COVID-19 infections and percentage of prehospital advanced airway insertions by month

A comparison of the prehospital interventions of the groups is presented in Table 3. First responder response interval was defined as the time from call received until the arrival of the first responder to the scene. EMS response interval was defined as the time from call received until the arrival of the ALS team to the scene. Scene time interval was defined as the time from the arrival of the ALS team until the departure of the ALS team from the scene. After initial prehospital resuscitation, the patients who were still in cardiac arrest were either pronounced dead at scene or transported and received CPR en route to the ED. All patients who had ROSC were transported to the ED. Figure 2 illustrates a decrease in advanced airway insertion after the peak of the COVID-19 outbreak in 2020. However, there were no significant differences in the prehospital ROSC between the groups.

**Table 3:** Prehospital care management in EMS utilized patients of pre-COVID-19 and COVID-19 outbreak groups

Characteristics	Total (***N*** = 515) ***n*** (%)	Pre-COVID-19 outbreak (2019) (***n*** = 254) ***n*** (%)	COVID-19 outbreak (2020) (***n*** = 261) ***n*** (%)	***P*** value
**First responder dispatch (missing = 1)**	330 (64.2)	180 (70.9)	150 (57.7)	0.002
**First responder response interval (median, IQR) (missing = 186)**	7.2 (4.1–11.5)	5.31 (3.2–9.3)	10 (6–14)	< 0.001
**First responder response within 4 min (missing = 186)**	63 (19.1)	52 (29.1)	11 (7.3)	< 0.001
**EMS response interval (median, IQR) (missing = 6)**	10 (7–15)	10 (7–14)	10 (7–15)	0.563
**EMS response within 8 min, (missing = 6)**	128 (25.1)	56 (22.5)	72 (27.7)	0.176
**Scene time interval (median, IQR) (missing = 9)**	16 (10–24)	17 (10.4–24.7)	16 (10–23)	0.238
**First arrest rhythm** **(missing = 25)**				0.083
Asystole	298 (60.8)	145 (60.4)	153 (61.2)	
Ventricular fibrillation	43 (8.8)	18 (7.5)	25 (10)	
Ventricular tachycardia	2 (0.4)	2 (0.8)	0 (0)	
Pulseless electrical activity	75 (15.3)	41 (17.1)	34 (13.6)	
Unknown shockable rhythm	14 (2.9)	11 (4.6)	3 (1.2)	
Unknown non-shockable rhythm	15 (3.1)	5 (2.1)	10 (4)	
Unknown	43 (8.8)	18 (7.5)	25 (10)	
**First arrest rhythm groups (missing = 25)**				0.554
Shockable rhythm	59 (12.0)	31 (12.9)	28 (11.2)	
Non-shockable rhythm	388 (79.2)	191 (79.6)	197 (78.8)	
Unknown	43 (8.8)	18 (7.5)	25 (10)	
**Prehospital defibrillation**	106 (20.6)	51 (20.1)	55 (21.1)	0.780
**Mechanical CPR (missing = 30)**	241 (49.7)	114 (47.5)	127 (51.8)	0.340
**Prehospital advanced airway (missing = 30)**	307 (63.3)	177 (73.8)	130 (53.1)	< 0.001
**Prehospital intubation**	278 (57.3)	160 (66.7)	118 (48.2)	< 0.001
**Prehospital supraglottic airway devices**	29 (6.0)	17 (7.1)	12 (4.9)	0.302
**Prehospital drug administration (missing = 31)**	363 (75.0)	190 (79.5)	173 (70.6)	0.024
**ROSC at scene (missing = 30)**	99 (20.4)	54 (22.5)	45 (18.4)	0.259
**ROSC at scene in shockable-rhythm group (*n*** **= 59)**	12 (20.3)	8 (25.8)	4 (14.3)	0.272
**Pronounced dead at scene**	135 (26.2)	60 (23.6)	75 (28.8)	0.178

*Abbreviations*: *COVID-19* coronavirus disease 2019, *CPR* cardiopulmonary resuscitation, *EMS* emergency medical service, *ROSC* return of spontaneous circulation

Table 4 shows the hospital outcomes of the pre-COVID-19 and COVID-19 groups. There were 39 (5.6%) missing data on sustained ROSC at ED, 46 (6.7%) on survival to ICU/ward admission, and 53 (7.7%) on survival to discharge.

**Table 4:** Outcomes of ED of pre-COVID-19 and COVID-19 outbreak groups

Characteristics	Total (***N*** = 691) ***n*** (%)	Pre-COVID-19 outbreak (2019) (***n*** = 341) ***n*** (%)	COVID-19 outbreak (2020) (***n*** = 350) ***n*** (%)	***P*** value	Crude odds ratio (95% CI)	Adjusted odds ratio (95% CI)
**All patient**						
Sustained ROSC at ED (missing = 39)	209 (32.1)	109 (33)	100 (31.3)	0.589	0.91 (0.66–1.27)	1.01 (0.68–1.49)
Survival to ICU/ward admission (missing = 46)	154 (23.9)	91 (27.8)	63 (19.8)	0.017	0.64 (0.44–0.93)	0.78 (0.49–1.15)
Survival to discharge (missing = 53)	32 (5.0)	25 (7.7)	7 (2.2)	0.002	0.27 (0.12–0.64)	0.34 (0.15–0.95)
**In shockable rhythm patient (*n*** **= 85)**						
Sustained ROSC at ED (missing = 4)	29 (35.8)	17 (41.5)	12 (27.3)	0.079	0.61 (0.24–1.52)	0.63 (0.19–2.03)
Survival to ICU/ward admission (missing = 5)	26 (32.5)	17 (41.5)	9 (20.5)	0.069	0.39 (0.15–1.04)	0.46 (0.13–1.61)
Survival to discharge (missing = 6)	10 (12.7)	8 (19.5)	2 (4.5)	0.087	0.20 (0.04–1.03)	0.67 (0.09–4.88)

*Abbreviations*: *CI* confidence interval, *COVID-19* coronavirus disease 2019, *ED* emergency department, *ICU* intensive care unit, *ROSC* return of spontaneous circulation

## Discussion

This multicentered study used data from the Thai OHCA Network Registry to determine the impact of the COVID-19 outbreak on OHCA management and outcomes. Hyperlipidemia was more prevalent among the COVID-19 outbreak group. The investigation revealed that the first-responder response interval for patients with OHCA in the participating areas was significantly longer during the COVID-19 outbreak than in the pre-COVID-19 period. In contrast, the EMS response intervals for the 2 periods were the same. The outbreak did not significantly affect the bystander CPR rate. However, the COVID-19 group had significantly lower rates for first responder dispatch, prehospital advanced airway insertion, prehospital CPR drug administration, and survival to hospital discharge.

Hyperlipidemia is one of the well-established cardiovascular risk factors. There was no study reporting the difference proportion of cardiac arrest patients with hyperlipidemia during the COVID-19 outbreak. In addition, there were no differences in other cardiovascular risk factors such as diabetes and hypertension in our study. Further studies are required to investigate the cause and association between hyperlipidemia and OHCA patients during the COVID-19 outbreak.

A shortage of PPE and the use of a volunteer-based first-responder system had severe effects: a decreased first-responder dispatch rate and a longer response interval. Without adequate PPE and charity subsidies for volunteer BLS ambulances during the economic depression, BLS teams were reluctant or even refused to accept missions with risks of COVID-19 infection, especially cases of OHCA in which aerosol-generating procedures were inevitable. This was a direct consequence of our particular low-resource EMS system.

However, the EMS response interval was unchanged. The hospital-based ALS teams were made up of medical personnel who worked in the ED during their shift and were prepared for EMS missions. Interestingly, no change in the EMS response interval contradicts studies in Singapore, Taiwan, and 5 Western countries. Their EMS response intervals increased regardless of the EMS system in use [10, 25, 26].

The COVID-19 outbreak did not significantly affect the rate of bystander CPR in the study. We speculate that this was partly because most of the OHCA cases during the lockdown occurred at home, where family members willingly performed CPR. A meta-analysis in 2021 that gathered data primarily from developed countries identified a slight but significant increase in the bystander CPR rate during the outbreak period (44.1% vs 46.2%) [27]. In addition, a dispatcher would advise hands-only dispatcher-assisted CPR instead of conventional CPR to encourage the bystanders to perform chest compression in the outbreak period. The purpose of this changed protocol was to encourage bystanders to perform chest compression during the outbreak period.

The study found that the prehospital intubation rate decreased during the COVID-19 outbreak. One of the 3 study sites launched a protocol requiring EMS personnel to swiftly transport patients to the ED and avoid prehospital advanced airway insertion. Other personnel with full PPE protection would then handle intubation, endotracheal tubes could be inserted in a controlled environment, and well-equipped airway devices such as video laryngoscopes were on hand. Moreover, rather than intubating patients with OHCA and presumed COVID-19 themselves, the EMS personnel at all 3 study sites would have found it less threatening to transport them to the ED where other personnel would manage the patient.

In contrast, the rate of advanced airway placement in other countries, especially supraglottic airway devices, increased significantly [27, 28]. A study from Korea demonstrated a decrease in the rate of tracheal intubation but a dramatic increase in the utilization of supraglottic airway devices [28]. Our study showed a considerably lower rate of SGA utilization both before and during the outbreak. This is because the cost of SGAs was significantly higher than the cost of endotracheal tubes, leading to limited use of SGAs in our low-resource setting.

It is still unclear why the rate of prehospital CPR drug administration decreased during the COVID-19 outbreak period. No change was made to the protocol for administering CPR drugs by any of the 3 study sites. Data on prehospital drug administration have rarely been reported in the literature. A study from Korea did not demonstrate a change in prehospital epinephrine administration [28]. We assume that our EMS teams preferred to quickly transport patients with OHCA to the ED for definite airway management, leaving intravenous access and drug administration as the second priority. This preference would have been strong when hospitals were minutes from the scenes.

As in other parts of the world [27], our study found a decreased survival rate to discharge during the COVID-19 outbreak. The decline was possibly due to the combined effects of a longer first-responder response interval, a lower first-responder response rate, less prehospital advanced airway insertion and drug administration, and overburdened EDs and intensive care units. These disruptions to the chain of survival undoubtedly impaired the chances of survival of the cardiac arrest patients. Apart from the mortality rate of COVID-19 itself, this worsening outcome reflected the consequences of prehospital care protocol changes and the effect of the low-resource EMS system on the first responder response.

To improve the survival chances of patients with OHCA during the COVID-19 outbreak, we recommend supplying both first-responder and hospital EMS teams with adequate supplies of PPE and resuscitation equipment. Surge capacity must also be developed and maintained to handle mild cases of COVID-19, thereby sparing first responders for actual emergency patients. Additionally, in the face of limited resources, EMS providers must consider comprehensively to determine whether resuscitation efforts should be limited.

## Limitations

This study has several limitations. Firstly, there were missing data, as expected with any retrospective observational study. Survival outcomes and important variables were sometimes not able to be obtained when patients were transported to hospitals that did not participate in the registry. Differences in the pre-COVID-19 and COVID-19 periods should be interpreted with caution, depending on the magnitude of missing data for each variable. In addition, time to prehospital defibrillation, as one of the core process measures, was not presented in the study due to uncertainty of data. This could be a potential confounder to survival outcomes. Secondly, each study site had slightly different EMS systems and OHCA protocols. These differences might have contributed to the heterogeneity of the outcomes. Moreover, only 3 sites were participating in the Thai OHCA Network Registry during the study period. All were tertiary academic hospitals that would have more resources than nonacademic hospitals. Therefore, this study might have underestimated the deleterious effects of the COVID-19 outbreak on national outcomes. Lastly, despite being categorized in the low-resource setting, Thailand is among the upper-middle-income countries. There might be great differences in EMS resources, EMS performance, and survival outcomes between Thailand and other lower income countries.

## Conclusions

In conclusion, there was a significant decrease in the survival rate to hospital discharge of out-of-hospital cardiac arrest patients during the COVID-19 outbreak compared to the pre-COVID-19 period in Thailand. This decline may represent the outcomes of low-resource EMS systems.

## Data Availability

The data that support the findings of this study are available from Thailand’s OHCA Network Registry. Restrictions apply to the availability of these data, which were used under license for this study. Data are available from the authors with the permission of Thailand’s OHCA Network Registry.
